# Establishing elements of a synthetic biology platform for Vaccinia virus production: BioBrick™ design, serum-free virus production and microcarrier-based cultivation of CV-1 cells

**DOI:** 10.1016/j.heliyon.2017.e00238

**Published:** 2017-02-04

**Authors:** Shuchang Liu, Ludmila Ruban, Yaohe Wang, Yuhong Zhou, Darren N. Nesbeth

**Affiliations:** a Department of Biochemical Engineering, University College London, Bernard Katz Building, London WC1E 6BT, UK; b Barts Cancer Institute, Queen Mary University of London, London EC1 M 6BQ, UK

**Keywords:** Biological sciences, Biochemistry, Bioengineering, Cell biology, Microbiology History

## Abstract

Vaccinia virus (VACV) is an established vector for vaccination and is beginning to prove effective as an oncolytic agent. Industrial production of VACV stands to benefit in future from advances made by synthetic biology in genome engineering and standardisation. The CV-1 cell line can be used for VACV propagation and has been used extensively with the CRISPR/Cas9 system for making precise edits of the VACV genome. Here we take first steps toward establishing a scalable synthetic biology platform for VACV production with CV-1 cells featuring standardised biological tools and serum free cell cultivation. We propose a new BioBrick™ plasmid backbone format for inserting transgenes into VACV. We then test the performance of CV-1 cells in propagation of a conventional recombinant Lister strain VACV, VACVL-15 RFP, in a serum-free process. CV-1 cells grown in 5% foetal bovine serum (FBS) Dulbecco’s Modified Eagle Medium (DMEM) were adapted to growth in OptiPRO and VP-SFM brands of serum-free media. Specific growth rates of 0.047 h^−1^ and 0.044 h^−1^ were observed for cells adapted to OptiPRO and VP-SFM respectively, compared to 0.035 h^−1^ in 5% FBS DMEM. Cells adapted to OptiPRO and to 5% FBS DMEM achieved recovery ratios of over 96%, an indication of their robustness to cryopreservation. Cells adapted to VP-SFM showed a recovery ratio of 82%. Virus productivity in static culture, measured as plaque forming units (PFU) per propagator cell, was 75 PFU/cell for cells in 5% FBS DMEM. VP-SFM and OptiPRO adaptation increased VACV production to 150 PFU/cell and 350 PFU/cell respectively. Boosted PFU/cell from OptiPRO-adapted cells persisted when 5% FBS DMEM or OptiPRO medium was observed during the infection step and when titre was measured using cells adapted to 5% FBS DMEM or OptiPRO medium. Finally, OptiPRO-adapted CV-1 cells were successfully cultivated using Cytodex-1 microcarriers to inform future scale up studies.

## 1 Introduction

Vaccinia virus (VACV) is an enveloped, brick-shaped particle typically 300 × 240 × 120 nm containing a double stranded DNA genome which, for the Lister strain (Garcel et al., 2007), is 189.4 kilobase-pairs (kb) in size, encoding up to 201 open reading frames (ORFs). VACV is arguably one of the most effective biotechnological tools in history by dint of the fact human antibodies raised against VACV tend also to recognise smallpox epitopes. VACV was used to eradicate smallpox via a global vaccination programme carried out by the World Health Organization (WHO) between 1966 and 1980 (Fenner et al., 1988).

VACV has also been used as a molecular biology tool to effect high-level transgene expression in mammalian cells, due in part to its ≈25 kb capacity for accommodating recombinant DNA (Mackett and Smith, 1986; Hruby, 1990; Guse et al., 2011). Molecular biology techniques developed in this area have also enabled construction of a wide range of recombinant VACV vaccines in which selected epitopes or payloads are defined by recombinant DNA.

Recombinant VACV has been developed as an effective live vaccine against viral, bacterial and oncological diseases (Hruby, 1990; Lee et al., 1994; Timiryasova et al., 1999; Zhang et al., 2007), due to its ability to elicit vigorous antibody and T-cell mediated responses. Hiley et al. (2010) and Tysome et al. (2011) have also demonstrated the effectiveness of recombinant Lister strain VACV in targeting hypoxic tumours in human head and neck cancer.

Mass production of VACV for smallpox eradication was achieved by harvesting virus from lesions brought about by infection of live animals (Fenner et al., 1988). However, this method brought significant risk of contamination with microbiological agents and was superseded by viral propagation in embryonated hen eggs (EAEMP 2002). Monath et al. (2004) investigated the use of MRC-5 cells to produce the New York City Board of Health (NYCBH) VACV strain for a Phase I clinical trial as a smallpox vaccine. As a human diploid cell line, MRC-5 has a finite *in vitro* life span that limits capacity for long-term cultivation . Large-scale VACV production using diploid cell lines can be difficult as such cells typically do not grow well on microcarriers (Barrett et al., 2009).

At laboratory-scale, scale-out strategies, such as roller bottles, T-flasks and the Nunc™ Cell Factory™, are commonly used to cultivate adherent cells for propagation of VACV. However, methods that can be scaled up, as opposed to scaled out, are the ideal solution for increasing the level of production, predictability and affordability for widespread application of VACV-based therapies. Toward this aim Bleckwenn et al. (2005) used HeLa S3 cells grown on microcarriers, at 1.5L scale, in a hollow fibre perfusion bioreactor setup to propagate VACV.

Viral vaccine production in media supplemented with bovine serum has been discouraged by regulatory authorities such as the Food and Drug Administration (FDA), brings high variability between serum batches and can lead to variations in product yield and quality. Undefined components in serum may also provide a route for adventitious agent contamination. Bioprocesses that are serum-free and animal derived component free (ADCF) are now sought in order to reduce the contamination risk, ease the downstream processing artefacts and promote robustness and reliability for the production of VACV. Previous attempts to grow CV-1 cells in serum-free media (Steimer et al., 1981) replaced serum with other animal-derived products so did not remove routes for adventitious agent contamination.

Synthetic biology aims to render biological phenomena easier to engineer (Ye and Fussenegger 2014). An inevitable consequence of this aim is that biology becomes easier to manufacture. When applied to VACV production, and its exploitation in areas such as gene therapy and oncotherapeutics, synthetic biology offers the prospect of rapid design and assembly of viral payloads using interoperable tools, such as BioBrick™-formatted plasmids (Shetty et al., 2008), compatible with repositories containing thousands of components. Synthetic DNA is now also being used to construct large segments of eukaryotic genomes (Dymond et al., 2011) and construction of human artificial chromosomes (Kononenko et al., 2015) is now an established approach in gene therapy research.

Vero cells are commonly used for VACV propagation and have been investigated in terms of their VACV production during cultivation in serum-free media (Mayrhofer et al., 2009), and on microcarriers (Monath et al., 2004). The CV-1 cell line is more often used for VACV titration (Schweneker et al., 2012) but recently multiple reports have been published demonstrating the use of the Cas9 nuclease/clustered regularly interspaced short palindromic repeats (Cas9/CRISPR) system to edit VACV genomes during CV-1 based virus propagation (Yuan et al., 2015a; Yuan et al., 2015b; Yuan et al., 2016a; Yuan et al., 2016b). The Cas9/CRISPR system enables precise, multiple edits of a genome to be made in parallel and has had a huge impact in the field of synthetic biology and beyond.

Because Cas9/CRISPR tools for VACV have been established in CV-1 cells, in this study we take the following first steps toward establishing a CV-1-based synthetic biology platform for VACV production: i) we propose a BioBrick™-formatted plasmid backbone for VACV genome engineering, ii) we retrofit VACV production in CV-1 cells from serum-containing media to serum-free media, iii) for serum-free adapted CV-1 cells, we measure growth performance and viral productivity during T flask cultivation and finally iv) we measure growth of serum-free adapted CV-1 cells in a microcarrier-based cultivation platform.

## 2 Material and methods

### 2.1 Cell cultivation

CV-1 cells, product CCL-70™ from American Type Culture Collection (ATCC), were grown in High Glucose Dulbecco’s Modification of Eagle’s Medium (DMEM) from PAA Laboratories (Pasching, Austria), supplemented with 5% v/v foetal bovine serum (FBS) from batches A10409-1728 and A15112-2026 for three passages prior to this study. Cells were passaged twice weekly in T flasks and seeded at 1 × 10^4^ cells/cm^2^ for growth in 5% FBS DMEM and serum-free OptiPRO and 2 × 10^4^ cells/cm^2^ for growth in serum-free VP-SFM medium. Serum-free media was supplemented with GlutaMAX to 4 mM and detached by treatment with TrypLE Select. All materials were sourced from Life Technologies, New York, USA, unless otherwise stated.

### 2.2 Cell banking and revival

Cells adapted to growth in 5% FBS DMEM were cryopreserved in 90% FBS plus 10% v/v dimethyl sulfoxide (DMSO) from Sigma-Aldrich (Ayrshire, UK). Cells adapted to growth in serum-free media were frozen in a v/v mixture of; 45% fresh growth medium, 15% 2 day, 15% 3 day and 15% 4 day conditioned medium plus 10% DMSO and 0.1% v/v methylcellulose (Sigma). Cells were suspended in the cryopreservation medium at 3–5 × 10^6^ cells/mL and transferred to 2 mL screw cap cryopreservation tubes (Eppendorf Ltd, Stevenage, UK) for storage in the liquid phase of a liquid nitrogen Dewar (Part No. 9902130, Statebourne Cryogenics, Tyne & Wear, UK). For revival, cryopreservation tubes were removed from liquid nitrogen and thawed in a SUB14 water bath at 37 °C (Grant Instruments, Cambridge, UK). Upon thawing, cells suspended in cryopreservation solution were diluted to a volume of 8 mL and centrifuged at 1300 rpm for 3 min. The supernatant was withdrawn and cell pellet was resuspended in 8 mL pre-warmed OptiPRO and transferred to a T 25 flask and incubated at 37 °C, 5% CO_2_ in an MCO-19AIC incubator (Sanyo, Gunma, Japan).

### 2.3 Counting cells cultivated using T flasks

Cells were detached from flask surfaces using standard trypsin treatment. Total cells in suspension were then counted using a TC10™ Automated Cell Counter (Bio-Rad, Hercules, USA) according to manufacturer’s instructions (document PN10016620 Rev B). Total viable cell counts in suspension were performed using standard trypan blue dye exclusion. Cells were stained with 0.4% trypan blue (#T8154, Sigma-Aldrich, Aryshire, UK) and counted using an Improved Neubauer haemocytometer (Hawksley, Lancing, UK) within three minutes of staining.

### 2.4 Adaptation to serum free media

Cells were grown in 10% FBS DMEM to a density of 1.3 × 10^5^ cells/cm^2^ in a T-25 flask (3.25 × 10^6^ cells total). Cells were then harvested into a total volume of 8 mL growth media mix, containing, for each round of adaptation; 6 mL, 4 mL, 2 mL, 0.8 mL and finally zero mL 10% FBS DMEM made up to 8 mL with serum-free media before further passaging. OptiPRO or VP-SFM brands of serum-free medium were used, as indicated in Fig. 2.

### 2.5 Cell growth kinetics in T flasks

Average cell growth rate (cells/cm^2^/day) was calculated using Eq. (1),

(1) $$\text{Cell}\;\text{ growth}=\frac{{\text{C}}_{\text{H}}-{\text{C}}_{\text{S}}}{\text{D}}$$

-where C_H_ is total cell density (cells/cm^2^) at harvest; C_S_ is the total cell density (cells/cm^2^) at seeding and D is culture duration (days). Cell Recovery Ratio (CRR) under complete serum free conditions was calculated using Eq. (2).

(2) $$\text{CRR}\%=100\%-\frac{{\text{N}}_{\text{F}}}{{\text{N}}_{\text{T}}}\times 100\%$$

-where N_F_ is total number of detached cells 24 h post seeding; N_T_ is total number of cells seeded (cells/cm^2^). Specific growth rate, μ (h^−1^) was based on Eq. (3).

(3) $$\mu \text{t}=\text{ln}\frac{{\text{x}}_{\text{n}}}{{\text{x}}_{0}}$$

Where x_0_ is starting total cell concentration per mL; t is the time of sampling in hours; x is total cell concentration per mL after t hours. A plot of ln x versus time (see Eq. (1)) gives a straight-line plot with μ as the slope. Cell doubling time, DT (hours) was calculated using Eq. (4), where μ_max_ is the maximum specific growth rate during the exponential phase, hour^−1^.

(4) $$\mathrm{DT}=\frac{\ln2}{\mu_{\max}}$$

### 2.6 Virus propagation

A single virus stock was used throughout this work. The Lister VACV, VACVL-15 RFP, has been propagated historically at Queen Mary University of London and encodes a red fluorescent protein (RFP) as reporter. A titre of 6.68 × 10^8^ PFU/mL was determined for this stock using the procedures described below and with CV-1 cells used for titration. Aliquots of virus for infection were diluted with the required volume of growth media. Virus solutions were added drop wise to cells at a density of 3–5 × 10^5^ cells/well in a 6-well plate at a multiplicity of infection (MOI) of 0.1. After the indicated time period, infected ‘propagator’ cells were harvested using a cell scraper for virus release by cell disruption.

### 2.7 Virus release from cells

Suspensions of cells infected for virus propagation were frozen in a −80 °C freezer for 30 min, thawed in a 37 °C water bath for 4 min and vortexed for 10 seconds. This freeze-thaw-vortex cycle was repeated three times and the resultant disruptate containing cell debris and released virus particles used for virus titration with no further purification.

### 2.8 Virus infection of target cells for titration

Median tissue culture infective dose (TCID_50_) was determined using CV-1 cells as indicator cells. Disruptates, containing cell debris and viruses, were serially diluted in 96-well plates containing cells adapted to, and grown in, 5% FBS DMEM unless otherwise stated. Cytopathic effect (CPE) was scored by light microscopy six days post infection. The Reed-Muench procedure (1938) was used to calculate TCID_50_ values, which were converted to PFU/cell using Eq. (5).

(5) $$\text{PFU}/\text{Cell}=\frac{0.69}{\text{TCID}50\text{value}\times \text{Vs}\times {\text{N}}_{\text{I}}}$$

-where Vs is the volume of sample used to infect the first row of the 96-well titration plates, mL; N_I_ is number of cells at infection.

### 2.9 Growth on microcarriers of CV-1 cells adapted to OptiPRO medium

#### 2.9.1 Pre-treatment of vessels and microcarriers

Cultivation of OptiPRO-adapted CV-1 cells adhered to Cytodex-1 microcarriers (GE Healthcare, Westborough, USA) was performed using a Techne MCS-104L 250 mL spinner flask setup (Bibby Scientific Ltd, Staffordshire, UK). All procedures were performed in a Level 1 laminar flow biological safety cabinet (BSC) unless otherwise stated. Spinner flasks were first prepared for use by siliconisation of the flask interior surface and impellers using Sigmacote (SL2, Sigma-Aldrich, USA) in accordance with manufacturer’s instructions. When the siliconisation procedure was complete the impeller system was assembled within the 250 mL spinner flask and autoclaved using a cycle of 20 min at 121 °C.

A 0.5L pyrex Duran bottle was also coated with silicon using the Sigmacote and following manufacturer’s instructions. Briefly, 100 mL of Sigmacote were poured into to the 0.5L pyrex Duran bottle which was swirled until all the interior surface received a covering of Sigmacote. Remaining Sigmacote was decanted and the bottle was autoclaved, dried in a fume hood overnight then rinsed with Milli-Q water to remove any siliconisation by-products before use.

The required mass of Cytodex-1 microcarriers (17-0448-01, GE Healthcare, Sweden) was added to the siliconised Pyrex Duran bottle. For every gram of dry microcarriers 100 mL of pH 7.4 PBS, free of calcium and magnesium ions (Life Technologies, Paisley, UK), was added. The bottle was left to stand over night to achieve complete swelling and hydration of the microcarriers. The next day the PBS used to hydrate the microcarriers was gently decanted and replaced with 50 mL fresh PBS for every gram of wet microcarriers. The microcarrier slurry was then sterilized by autoclaving (121 °C, 20 min). Prior to use, the sterilized microcarriers were allowed to cool and settle. Upon cooling the supernatant was gently decanted and replaced with 50 mL fresh OptiPRO medium for every gram of wet microcarriers. This OptiPRO/microcarrier slurry was allowed to settle and the OptiPRO supernatant was gently decanted then replaced with 100 mL fresh OptiPRO for every gram of wet microcarriers.

#### 2.9.2 Mixing cells and microcarriers

30 mL of the microcarrier/media slurry was transferred to the spinner flask. 70 mL of a suspension of OptiPRO-adapted CV-1 cells in OptiPRO was then added to the spinner flask to achieve a microcarrier concentration of 3 g/L and a cell concentration of 3.4 × 10^5^ cells/mL, corresponding to 26 cells/microcarrier. The spinner flask was then placed on the MCS-104L stirrer device inside an MCO-19AIC incubator (Sanyo, Gunma, Japan) at 37 °C and 5% CO_2_. The mixture of cells and microcarriers was subjected to intermittent agitation at 30 RPM for 30 seconds every 30 min for the first two days of cultivation after which continuous 30 RPM agitation was used for the third day and then 35 RPM for the remainder of the cultivation experiment. Approximately 70% of the volume of culture medium was replaced with fresh OptiPRO every 24 h by terminating agitation; allowing microcarriers to settle decanting supernatant and adding fresh OptiPRO.

#### 2.9.3 Removing CV-1 cell samples during microcarrier-based cultivation

The MCS-104L stirrer device was switched off and transferred, with the spinner flask, from the incubator to the BSC. The stirrer was set to agitate the spinner flask at 60 RPM and the cap from one side-arm port of the spinner flask was removed to allow withdrawal of a 0.2 mL sample from the culture. The MCS-104L stirrer device was then switched off and the entire setup returned to the incubator where incubation and agitation were resumed.

#### 2.9.4 Counting CV-1 cells during cultivation using microcarriers

Samples taken as above were typically transferred to a 1.5 mL Eppendorf tube and washed with 1 mL PBS. Microcarriers were allowed to settle and 1 ml supernatant was gently decanted; then a further 1 mL PBS wash performed. After the final decanting of supernatant 0.2 mL aqueous crystal violet solution (0.1% w/v Crystal Violet, 0.1 M citric acid, 0.1% v/v Triton X-100) was added to the microcarriers slurry and the mixed by pipetting up and down 25 times before the Eppendorf tube was transferred to an incubator set at 37 °C/5% CO_2_ for 1.5 h. This treatment causes cells to lyse and release stained nuclei. Typically the solution was diluted by addition of PBS. Released nuclei were counted using an Improved Neubauer haemocytometer (1080346, Heinz Herenz Medizinalbedarf GmbH, Hamburg, Germany) as an indicator of cell numbers.

## 3 Results and discussion

### 3.1 Proposal for a BioBrick™-based VACV plasmid tools

**Fig. 1:**
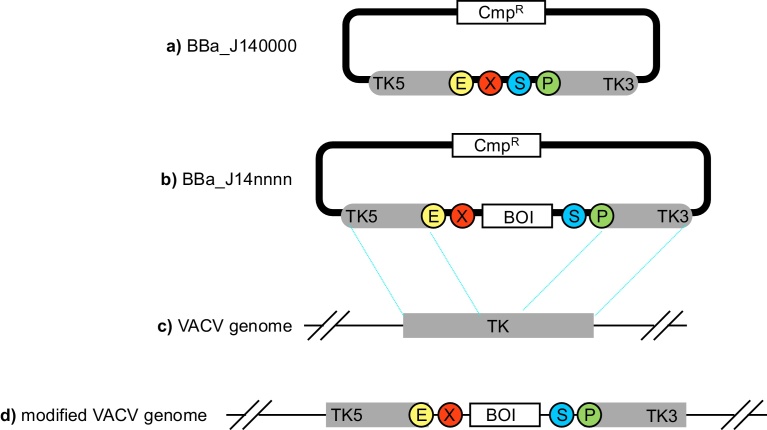
Design of a BioBrick™-formatted Vaccinia DNA tool. **a)** Overview of BBa_J140000, a proposed BioBrick™ plasmid backbone for VACV genome editing. The plasmid incorporates a chloroamphenicol resistance selection marker (Cmp^R^) and the BioBrick™ prefix: Eco RI (E), Xba I (X), and suffix: Spe I (S), Pst I (P). Not I sites are omitted for graphical brevity. The BioBrick™ cloning site is flanked by 5′ and 3′ ends of the TK locus for homologous recombination. **b)** A BioBrick™ of interest (BOI) can be cloned into BBa_J140000 using standard BioBrick™ assembly, resulting in a new plasmid, BBa_J14nnnn. **c)** Parallel cell infection with unmodified VACV and transfection with BBa_J14nnnn can then result in recombination for insertion of the BOI into the genome of replicating, progeny VACV. **d)** This insertion will result in a new, recombinant VACV.

The VACV genome is conventionally edited within mammalian cells by parallel viral infection and transfection with a plasmid. The plasmid typically encodes a transgene intended for insertion into the VACV genome is flanked by sequences identical to a VACV locus. Homologous recombination within the mammalian cell then directs insertion of the transgene at the intended VACV genome location (Fig. 1). Typically the locus encoding the ORF for thymidine kinase (TK) is used to target insertion of transgenes (Byrd and Hruby, 2004) as its disruption does not compromise virus replication in cells commonly used for virus propagation (Fig. 1). Plasmids designed for this purpose often feature a multiple cloning site flanked upstream and downstream by regions of homology with the VACV TK gene. However, such multiple cloning sites tend to be designed without consideration of any standards for DNA assembly.

The Registry of Standard Biological Parts is widely used by synthetic biologists and uses the BioBrick™ standard for plasmids and their assembly by ligation. The Registry, run by staff of the International Genetically Engineered Machines (iGEM) Foundation (Boston, USA), consists of a large plasmid library (Müller & Arndt, 2012) curated by users. All BioBrick™ formatted plasmids are inter-compatible so any plasmid designed in the BioBrick™ format is automatically compatible with the entire BioBrick™ registry.

For BioBrick™ compatibility a given DNA segment need only be flanked upstream by a defined sequence motif encoding, in order, EcoRI, NotI and XbaI restriction sites and downstream by, in order, SpeI, NotI and PstI sites (Canton et al., 2008; Shetty et al., 2011). These flanking sequences are known as the BioBrick™ prefix and suffix. BioBrick™ tools for virus design have been developed previously, such as BBa_K404129 that encodes a transgene expression cassette designed to be encapsidated by adeno-associated virus. Here we have designed a new BioBrick™ plasmid backbone, BBa_J140000, which features the BioBrick™ prefix and suffix flanked by 5′ and 3′ end regions of the VACV TK ORF. Any BioBrick™ of interest (BOI) can be inserted into this region of BBa_J140000 by ligation then subsequently into the VACV genome by co-transfection and recombination (Fig. 1).

### 3.2 Adaptation of CV-1 cells to serum free growth media

**Fig. 2:**
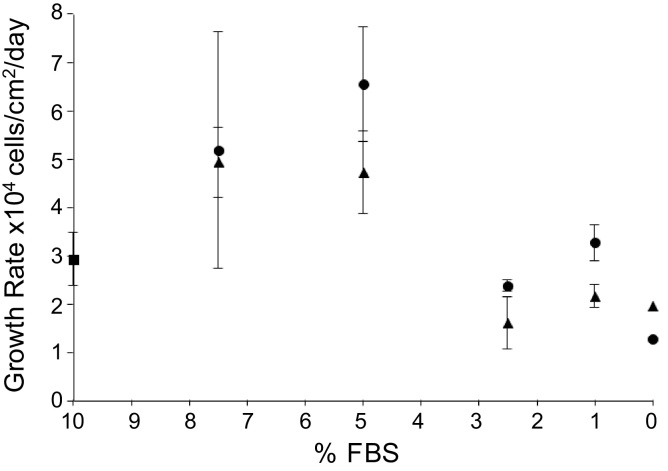
Average growth rates of CV-1 cells during stepwise adaption to serum-free media. Starting at 10% FBS DMEM (square) cells were harvested and re-plated in mixtures of serum-containing and serum-free medium to give the serially decreasing overall serum concentration indicated by the X-axis. VP-SFM (triangles) or OptiPRO SFM (circles) brands of serum free medium were used. Growth rates were determined as detailed in Methods.

CV-1 is a continuous cell line derived from *Cercopithecus aethiops* African green monkey kidneys by Jensen (1964). It is susceptible to several viruses including VACV and has been widely used for virus titration (Cho et al., 1970; Hiley et al., 2010). CV-1 cells were grown in a rich medium, 10% FBS DMEM, before stepwise adaptation to growth in the VP-SFM and OptiPRO brands of serum-free medium. The serum content (v/v) of the growth medium mix used during each round of adaptation was lowered to 7.5%, 2.5%, 1% and finally 0%. Fig. 2 shows the average growth rates observed when cells were first challenged with the decreased serum content media mix. Average CV-1 cell growth rate in 10% FBS DMEM was 2.93 ± 0.54 × 10^4^ cells/cm^2^/day. Average growth rates in 7.5% and 5% FBS media mixes, for both VP-SFM and OptiPRO, were increased compared to 10% FBS DMEM. Only 0% serum, pure VP-SFM or OptiPRO, resulted in initial growth rates lower than that for 10% FBS DMEM, with 1.97 × 10^4^ cells/cm^2^/day and 1.29 × 10^4^ cells/cm^2^/day respectively. Cells were then grown in VP-SFM or OptiPRO for another four passages before being considered as fully adapted to serum-free media.

### 3.3 Growth profiles of CV-1 cells adapted to serum-free media

**Fig. 3:**
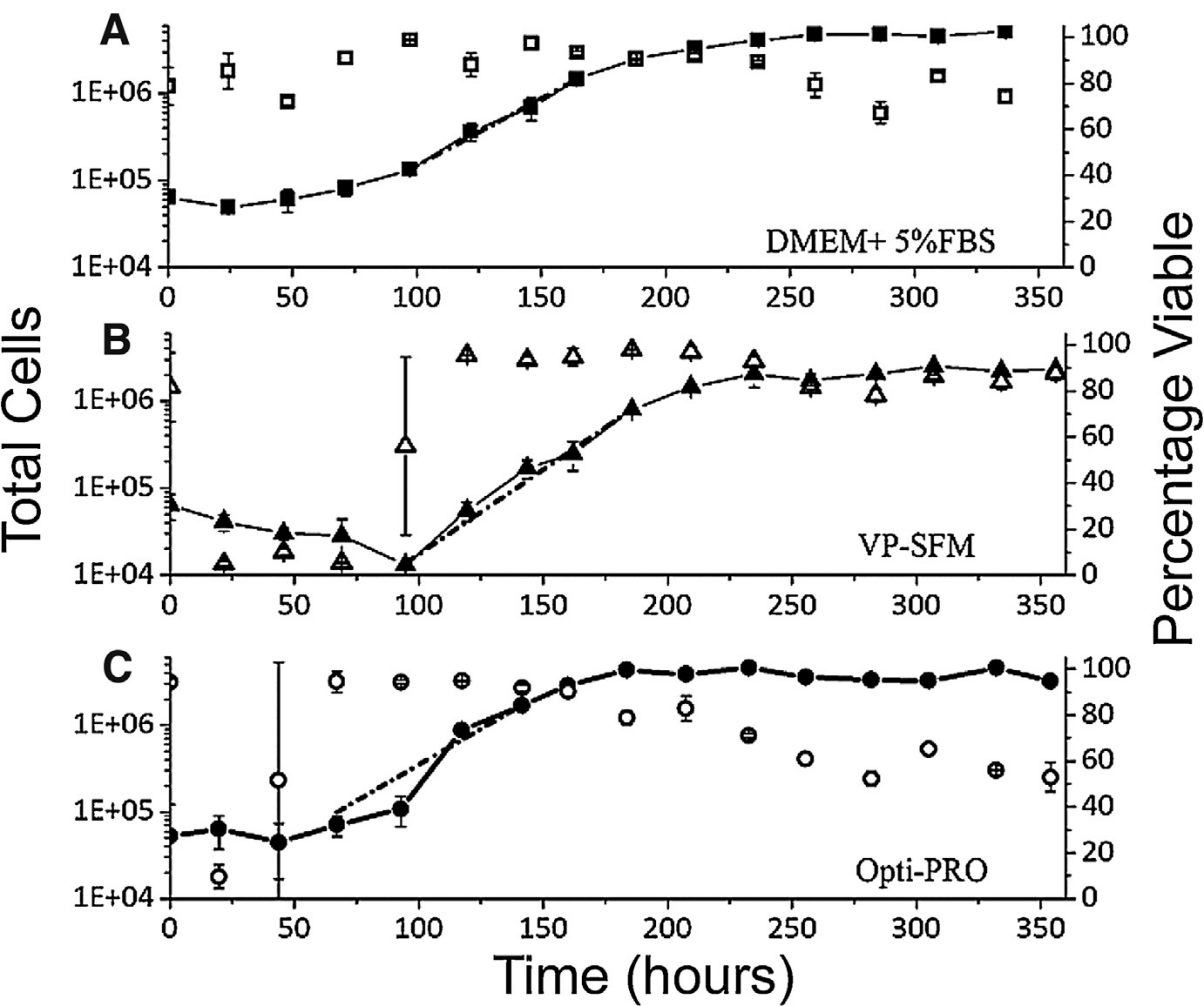
Growth of CV-1 cells adapted to serum-free media. Cells adapted to, and grown in, A) 5% FBS DMEM (squares), B) VP-SFM (triangles) and C) OptiPRO (circles) were seeded into T-25 flasks at the density indicated and their growth (closed symbols) and viability (open symbols) followed over 350 h. Total cells were detached by trypsinisation, counted and reseeded in fresh media at each time point, with 1–5% of material discarded after cell counting and viability assessment. Dashed line indicates the linear regression of cell growth during exponential phase used to calculate growth rates provided in Table 1. Error bars indicate standard deviation over n = 2 biological repeats.

Cells adapted to 5% FBS DMEM, VP-SFM and OptiPRO were seeded at 5 × 10^4^ cells/25 cm^2^ in T-25 flask and cell densities measured every 24 h for 350 h to observe lag, exponential and stationary phases (Fig. 3) and determine growth rate (Table 1). For cells adapted to 5% FBS DMEM (Fig. 3A), total cell numbers decreased over the first 24 h post-seeding. Growth increased after this with a specific growth rate of 0.035 h^−1^ observed, corresponding to a doubling time of 20.1 h. This is comparable to the doubling time of 22 h reported by Hagedorn et al. (1985) for CV-1 cells grown in medium with 5% v/v foetal calf serum (FCS). Cell growth slowed after 168 h, at a saturation density of 4.52 × 10^6^ cells/25 cm^2^. Saturation density of adherent cells on a solid surface is a potential indicator of microcarrier growth performance.

Cells adapted to VP-SFM (Fig. 3B), decreased rapidly in number over the first 95 h post-seeding, with viability as low as 4.9%. Cells then entered exponential growth, achieving 90% viability and a specific growth rate of 0.044 h^−1^ (15.9 h doubling time). Cells entered stationary phase 235 h post-seeding with saturation density of 2.15 × 10^6^ cells/25 cm^2^.

Growth of cells adapted to OptiPRO (Fig. 3C) lagged over the first 67 h post-seeding then grew exponentially, with a specific growth rate of 0.047 h^−1^ (14.8 h doubling time). These cells reached stationary phase at 192 h post-seeding, at a saturation density of 3.84 × 10^6^ cells/25 cm^2^. Notably, a significant decrease in viability was observed 24 h post-seeding. This increased to >90% at 67 h, remained above 90% until 200 (160) hours then declined to <60% at 354 h post-seeding.

Compared to cells adapted to grow in 5% FBS DMEM, cells adapted to VP-SFM and OptiPRO had lower saturation densities and significant drops either in cell number or viability in the first 96 h post-seeding. The absence of specific growth factors (Todaro et al., 1965; Vogel et al., 1980) or nutrients from certain serum-free media formulations may result in reduced shear resistance in mammalian cells (EL-Ensahsy et al., 2009). This could explain the extended lag phase growth of cells adapted to VP-SFM and OptiPRO compared to those adapted to 5% FBS DMEM (Fig. 3). VP-SFM and OptiPRO-adapted cells may have required a longer time period to recover from shear experienced over multiple rounds of detachment, resuspension and re-seeding during adaptation (Fig. 2).

### 3.4 CV-1 cell robustness to cryopreservation

Stable and reliable recovery from cryopreservation is a critical attribute of mammalian cells used for industrial production of biotherapeutics. Recovery ratio provides an indication of the effectiveness of a given formulation of cryopreservant media for storing cells under liquid nitrogen. Cells adapted to growth in 5% FBS DMEM, VP-SFM and OptiPRO were resuspended in cryopreservant media, as detailed in the Methods section above, containing methylcellulose as a protective agent only for CV-1 cells previously adapted to VP-SFM and OptiPRO (Waymouth and Vamum, 1976). After storage in liquid nitrogen for six months cells were revived and recovery ratios determined (Table 1). Recovery ratios of ≈97% were measured for cells grown in, and adapted to, both OptiPRO and 5% FBS DMEM. Cells adapted to VP-SFM had the lowest recovery ratio of ≈82%.

### 3.5 Vaccinia virus production by CV-1 cells adapted to grow in serum-free media

**Fig. 4:**
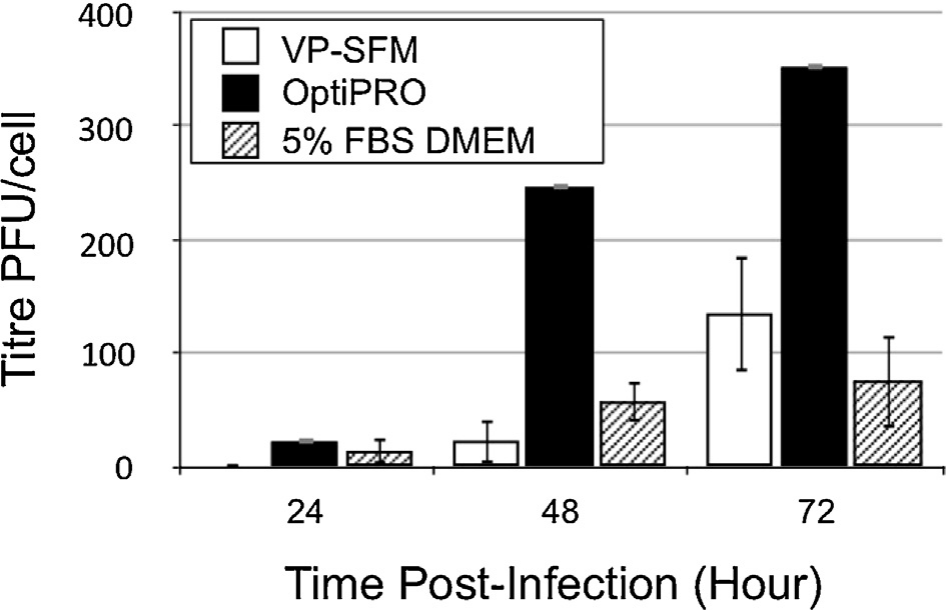
Viral productivity of CV-1 cells adapted for growth in serum free media. Viral titre productivity of CV-1 cells adapted for growth in VP-SFM (open bar), OptiPRO (black bars) and 5% FBS DMEM (striped bars). Cells at 80–95% confluence were infected with VACVL-15 RFP at MOI = 0.1 and virus liberated for titration at indicated times post-infection. Error bars indicate standard deviation over n = 3 biological repeats.

For production of VACV strain TSI-GSD-241 using MRC-5 cells for propagation, Wu et al. (2005) reported virus productivity of 77 PFU/cell when the cells were grown in 20% FBS DMEM and infected at an MOI of 0.1. We used a recombinant Lister strain VACV, VACVL-15 RFP, encoding a red fluorescent protein expression cassette payload. At an MOI of 0.1 we infected CV-1 cells (as ‘propagators’) adapted to, and cultivated in, 5% FBS DMEM, VP-SFM or OptiPRO. Infected cells were incubated at 37 °C with 5% CO_2_ for 24, 48 and 72 h post-infection after which virus was released and virus titre measured (Fig. 4) by infection of CV-1 cells adapted to, and cultivated in, 5% FBS DMEM as ‘targets’ cells. At 72 h post infection ‘propagator’ cells adapted to growth in OptiPRO achieved a titre of 352 PFU/cell, 4.6 fold higher than the titre achieved by cells adapted to 5% FBS DMEM and 2.6 fold higher than the titre achieved by cells adapted to VP-SFM.

### 3.6 Influence of media type and cell provenance on viral titre performance

It is not evident from Fig. 4 whether the enhanced titre performance of OptiPRO-adapted cells results from OptiPRO favouring virus infection events or OptiPRO exerting a selective pressure that favours CV-1 cells capable of high virus productivity. Furthermore, compatibility with a notional synthetic biology production platform for VACV manufacture would require multiple iterations of entirely serum-free propagation.

**Fig. 5:**
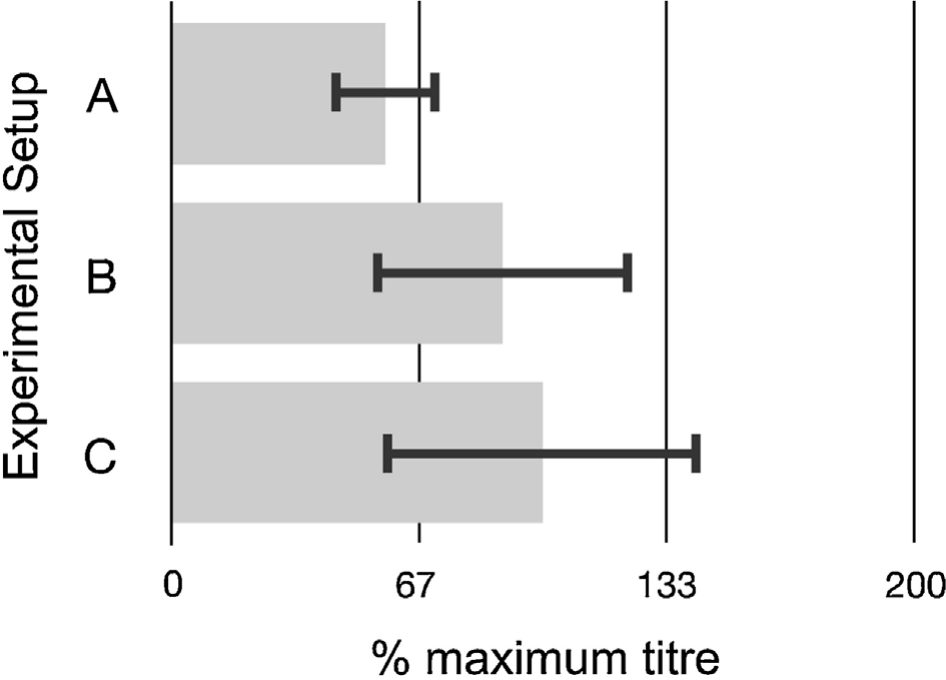
Influence of media and cell type on viral titre performance. As set out in Table 2, CV-1 cells were washed in PBS and re-immersed either in OptiPRO or 5% FBS DMEM, infected with VACV at MOI = 0.1 and 72 h post-infection progeny virus used to infect cells adapted to growth in OptiPRO in the presence of OptiPRO (Experimental setup A) or 5% FBS DMEM (Experimental setup B), or cells adapted to growth in 5% FBS DMEM in the presence of 5% FBS DMEM (Experimental setup C). Error bars indicate standard deviation over n = 3 biological repeats. As Experimental setup C was a repeat of the experiment performed to generate the data in Fig. 4 (72 virus harvest 72 h post-infection), the titre achieved with Experimental setup C was set as the 100% level.

As such we repeated the OptiPRO, 72 h post-infection harvest time experiment of Fig. 4 alongside two comparator experiments in an attempt to determine both the likely causative factors for the increased titre observed in Fig. 4 and the relative efficiency of an entirely serum-free round of propagation. Table 2 summarises our approach; cells adapted for growth in OptiPRO were grown to 95% confluence then washed twice with PBS before immersion either again in OptiPRO (Fig. 5A and C) or 5% FBS DMEM (Fig. 5B), immediately prior to infection. For titration, OptiPRO-adapted cells in the presence of OptiPRO (Fig. 5A) and 5% FBS DMEM-adapted cells in the presence of 5% FBS DMEM (Fig. 5B and C) were used as ‘targets’ for titre measurement.

Experiment C (Table 2) is a straight repeat of the conditions used in Fig. 4 (data in black bars, harvest 72 h post-infection) so the resultant titre was set as the 100% level for comparison with Experiments A and B (see Table 2, Fig. 5A and B). If the presence of OptiPRO media enhances VACV infection of CV-1 cells, then Experiment A could be expected to increase the titre achieved by Experiment C (see Table 2, Fig. 5A and C). This is not the case, with Experiment A yielding at best the same titre performance as Experiment C. If the presence of 5% DMEM enhances VACV infection of CV-1 cells, then Experiment B could be expected to increase the titre achieved by Experiment C (see Table 2, Fig. 5B and C). This is not the case, with Experiment B yielding at best the same titre performance as Experiment C.

Taken together, observations from Fig. 4, Fig. 5 are consistent with the enhanced titre observed for OptiPRO-adapted cells being due to the process of adaptation to OptiPRO media also exerting a post-infection phenotype of increased virus productivity. They also indicate that entirely serum-free rounds of VACV propagation, such as those likely to define industrial synthetic biology platforms, yield comparable titre performance to serum-containing processes and so are feasible.

### 3.7 CV-1 cell cultivation using OptiPRO and microcarriers

**Fig. 6:**
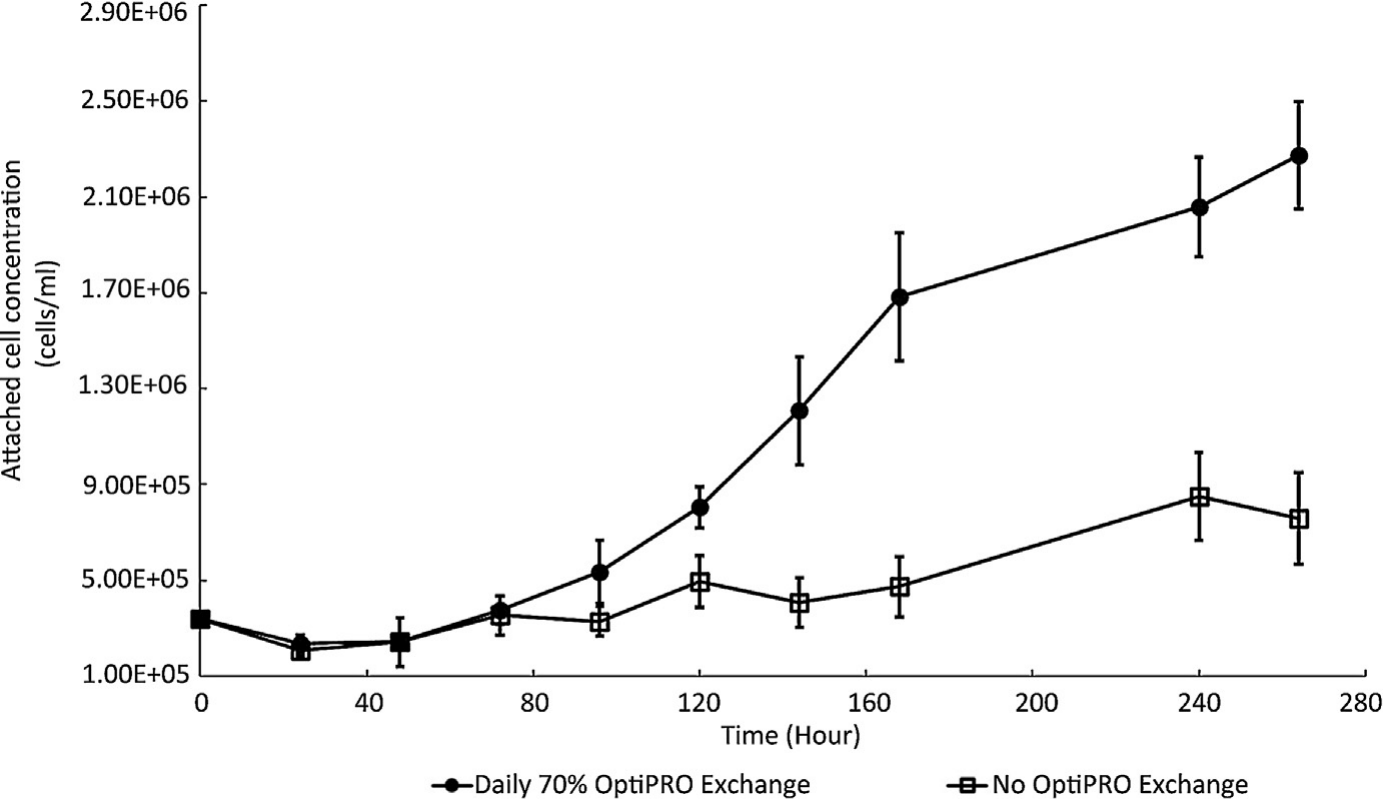
Cultivation of CV-1 cells adapted to OptiPRO media on microcarriers**.** Cells adapted for growth in OptiPRO were mixed with Cytodex-1 microcarriers at a starting ratio of 26:1 in 100 mL OptiPRO and agitated at 30–35 RPM over the indicated period. Cell concentrations were plotted for cultivation in which either 70% of the OptiPRO was changed every 24 h (black circles) or OptiPRO was unchanged throughout (open squares). Error bars indicate standard deviation over n = 3 biological repeats, P < 0.05.

We sought to determine if CV-1 cells adapted to OptiPRO could be cultivated using microcarriers (Fig. 6). We attempted cultivation using Cytodex-1 microcarriers and a Techne MCS-104L 250 mL spinner flask setup (Bibby Scientific Ltd, Staffordshire, UK) in which microcarrier suspensions were agitated by bulb–shaped glass impellers driven directly by a magnetic base. OptiPRO-adapted CV-1 cells were able to grow on micocarriers when approximately 70% of OptiPRO mediumwas changed daily and showed reduced growth when the OptiPRO made was unchanged over 270 h of cultivation (Fig. 6).

In the case of T-flask cultivation, VACV propagation involves two significant factors: virus number and cell numbers, which are summarised by the MOI. By contrast microcarrier-based VACV propagation presents three major factors; viruses, cells and microcarriers, and as such represents a complex investigation to identify productivity optima, such as those reported by Monath et al. (2004) for production of VACV from Vero cells grown using microcarriers and Bleckwernn et al. at (2005) for VACV production from HeLa cells. Such an investigation falls outside the scope of this study, which is to indicate the broad feasibility of the steps likely to define a future synthetic biology platform for VACV production.

## 4 Conclusions

We have proposed a new BioBrick™ plasmid backbone, BBa_J140000, which in effect makes every BioBrick™ in the Registry of Standard Biological Parts available for insertion into the TK locus of VACV without the need for bespoke cloning strategies. CV-1 cells adapted for growth in OptiPRO serum free medium exhibited elevated titre performance when grown using static culture. Mechanisms underlying the elevated titre are unclear but may result from selective pressure exerted by the adaptation process acting also to select for an unintended phenotype. These adapted CV-1 cells also showed promising growth characteristics on Cytodex-1 microcarriers.

Overall these results are consistent with the assertion that a standardised, serum-free, microcarrier-based synthetic biology platform for production of VACV is feasible. Cultivation in suspensions is inherently more scalable than cultivation on planar surfaces so further scale up of the platform proposed here should be investigated. Future work should include an investigation of the optimal conditions for VACV production from CV-1 cells grown on microcarriers, including the ratio of infecting virus to cells and microcarriers. Characterisation of virus quality should also be performed to assess factors such as the percentage of total virus particles that are plaque-forming as opposed to inactive.

## Declarations

### Author contribution statement

Shuchang Liu: Conceived and designed the experiments; Performed the experiment; Analyzed and interpreted the data; Contributed reagents, materials, analysis tools or data.

Ludmila Ruban: Contributed reagents, materials, analysis tools or data.

Yaohe Wang: Conceived and designed the experiments; Contributed reagents, materials, analysis tools or data.

Yuhong Zhou: Conceived and designed the experiments; Contributed reagents, materials, analysis tools or data.

Darren N. Nesbeth: Conceived and designed the experiments; Contributed reagents, materials, analysis tools or data.

### Competing interest statement

The authors declare no conflict of interest.

### Funding statement

This research did not receive any specific grant from funding agencies in the public, commercial, or not-for-profit sectors. The authors would like to thank the EPSRC (EP/I033270/1) for partial support of this work.

### Additional information

No additional information is available for this paper.

## Figures and Tables

**Table 1:** Performance of cells adapted to serum-containing and serum-free media types. Summary of performance data for cells adapted to growth in the different media types. Column 1 shows recovery from cryopreservation (recovery ratio). Column 2 provided growth rates of cells after adaptation to the indicated media type.

	1	2
Medium used for cell adaptation and growth	Recovery Ratio (% ± STD)	Specific Growth Rate μ (h^−1^)
VP-SFM	82.10 ± 3.59	0.044
OptiPRO SFM	97.31 ± 0.34	0.047
5%FBS DMEM	97.15 ± 0.72	0.035

**Table 2:** Dissecting effect of growth medium and cell-adaptation on viral infection and production. Experiment setup A: CV-1 cells adapted to growth in OptiPRO were washed in PBS, re-immersed in OptiPRO and infected with VACV at MOI = 0.1. 72 h post-infection, progeny virus from these cells was used to infect CV-1 cells adapted to growth in OptiPRO, in the presence of OptiPRO. Experiment setup B: CV-1 cells adapted to growth in OptiPRO were washed in PBS, immersed in 5% FBS DMEM and infected with VACV at MOI = 0.1. 72 h post-infection, progeny virus from these cells was used to infect CV-1 cells adapted to growth in 5% FBS DMEM, in the presence of 5% FBS DMEM. Experiment setup C: CV-1 cells adapted to growth in OptiPRO were washed in PBS, re-immersed in OptiPRO and infected with VACV at MOI = 0.1. 72 h post-infection, progeny virus from these cells was used to infect CV-1 cells adapted to growth in 5% FBS DMEM, in the presence of 5% FBS DMEM. Data generated from these experiments were plotted in Fig. 5. *Propagator: cells that are infected with virus for the purpose of harvesting virus particles from those cells. **Target: cells that have been infected with virus in order to establish a TCID_50_ as an indication of the titre of the virus solution used to infect the cells.

Experiment	A	B	C
Propagator cell*	Cells adapted to growth in OptiPRO	Cells adapted to growth in OptiPRO	Cells adapted to growth in OptiPRO
Media present during Propagator cell infection	OptiPRO	5% FBS DMEM	OptiPRO
Target cell**	Cells adapted to growth in OptiPRO	Cells adapted to growth in 5% FBS DMEM	Cells adapted to growth in 5% FBS DMEM
Media present during Target cell infection	OptiPRO	5% FBS DMEM	5% FBS DMEM
